# Disjoint and Functional Principal Component Analysis for Infected Cases and Deaths Due to COVID-19 in South American Countries with Sensor-Related Data

**DOI:** 10.3390/s21124094

**Published:** 2021-06-14

**Authors:** Carlos Martin-Barreiro, John A. Ramirez-Figueroa, Xavier Cabezas, Víctor Leiva, M. Purificación Galindo-Villardón

**Affiliations:** 1 Department of Statistics, Universidad de Salamanca, 37008 Salamanca, Spain; cmmartin@espol.edu.ec (C.M.-B.); jramirez@espol.edu.ec (J.A.R.-F.); pgalindo@usal.es (M.P.G.-V.); 2 Faculty of Natural Sciences and Mathematics, Universidad Politécnica ESPOL, Guayaquil 090902, Ecuador; joxacabe@espol.edu.ec; 3 School of Industrial Engineering, Pontificia Universidad Católica de Valparaíso, Valparaíso 2362807, Chile

**Keywords:** data science, disjoint and functional components, infectious diseases, k-means clustering, multivariate statistical methods, R software, SARS-Cov2, sensing and data extraction

## Abstract

In this paper, we group South American countries based on the number of infected cases and deaths due to COVID-19. The countries considered are: Argentina, Bolivia, Brazil, Chile, Colombia, Ecuador, Peru, Paraguay, Uruguay, and Venezuela. The data used are collected from a database of Johns Hopkins University, an institution that is dedicated to sensing and monitoring the evolution of the COVID-19 pandemic. A statistical analysis, based on principal components with modern and recent techniques, is conducted. Initially, utilizing the correlation matrix, standard components and varimax rotations are calculated. Then, by using disjoint components and functional components, the countries are grouped. An algorithm that allows us to keep the principal component analysis updated with a sensor in the data warehouse is designed. As reported in the conclusions, this grouping changes depending on the number of components considered, the type of principal component (standard, disjoint or functional) and the variable to be considered (infected cases or deaths). The results obtained are compared to the k-means technique. The COVID-19 cases and their deaths vary in the different countries due to diverse reasons, as reported in the conclusions.

## 1. Introduction

The COVID-19 pandemic has deteriorated the usual dynamics that rule the world [1]. Its impact on health and the worldwide economy is evident [2,3]. The number of people affected by the COVID-19 disease, the significant number of human lives that have been lost, and the consequences of the containment measures are urgent concerns that should be analyzed. This must be done in a technically responsible way to guarantee an effective response from the authorities of each country [4].

The Coronavirus (COVID-19) disease is caused by the novel severe acute respiratory syndrome coronavirus 2 (SARS-CoV-2) and was first identified in Wuhan City, Hubei Province, China, early in December 2019 [5]. COVID-19 arrived in South America at the end of February 2020 [4]. The coronavirus attacks some organs of the human body, but mainly the lungs [6]. In recent months, experts have detected the presence of some strains, as a result of the mutation of the virus, with higher rates of spread. As mentioned, the world has been greatly affected by the presence of this virus and governments are currently engaged in a mass vaccination process with the aim that people will be able to resume their activities in a normal way [4].

To confirm suspected cases of COVID-19, the real-time transcription-polymerase chain reaction (RT-PCR) test is applied, based on the detection of a certain amount of genetic fragments other than the virus in an individual [7]. Nevetheless, other diagnostic tools to detect COVID-19 cases have been proposed [8].

Proper planning of the response to an emergency is marked by the challenges of economic and political will [8]. However, the application and adaptation of statistical methods for data analysis that strengthen the interpretation of the information, taken from different points of interest, are actions that undoubtedly improve the quality of the response to the emergency [4]. These are actions that the citizens expect from their government authorities. As in other regions around the world, in South American countries, the number of infected people and deaths due to COVID-19 are increasing every day.

One popular multivariate statistical method is the principal component analysis (PCA), which allows for classification of variables or individuals [9,10]. The PCA has been used in several studies related to COVID-19 [11,12,13]. The objective of the present investigation is to apply the PCA to classify South American countries with respect to the number of infected and deaths due to COVID-19. We want to determine how the pandemic evolves in South American countries by using different multivariate methods, based on both matrix and functional approaches. These methods allow us to identify differences and similarities among the countries under study to understand the damage that the COVID-19 pandemic has caused them. The countries considered for this classification are: Argentina (ARG), Bolivia (BOL), Brazil (BRA), Chile (CHI), Colombia (COL), Ecuador (ECU), Peru (PER), Paraguay (PRY), Uruguay (URY), and Venezuela (VEN). We use modern techniques based on (i) standard PCA; (ii) disjoint principal component analysis (DPCA); and (iii) functional principal component analysis (FPCA). The full data set used in this investigation can be downloaded from https://ourworldindata.org/coronavirus (accessed on 12 June 2021). The data regarding the numbers (per million inhabitants) of infected cases and deaths are taken from 1 March 2020 to 15 March 2021. We design an algorithm that permits one to summarize the multivariate methods presented in this work to sense changes in the data by using a sensor and having an updated analysis.

The remainder of the paper is organized as follows: In Section 2, the fundamentals of the PCA, DPCA, and FPCA are provided. Section 3 and Section 4 apply the PCA, DPCA, and FPCA described in Section 2 to analyze the data collected. In Section 5, we discuss the results of the study and address the conclusions as well as ideas for future research.

## 2. Methods

### 2.1. Principal Component Analysis

The PCA is used in data matrices, with the measurements of a set of variables on a set of individuals being stored. The objective of the PCA is to reduce the original variables to a few latent variables (principal components) and to work in a reduced-dimension space that facilitates the interpretation. The principal components are obtained by solving an optimization problem defined as

(1) $$\begin{array}{ccc} \max\;(Z & = & w^{⊤}Vw),\\ \mathrm{subject}\mathrm{to}\;w^{⊤}w & = & 1, \end{array}$$

where $V=\left(1/n\right)X^{⊤}X$ is the sample matrix of covariances of the data matrix X (centered by columns), and the constraint $w^{⊤}w=1$ indicates that the vector w is of unitary norm, with X being a matrix with *n* individuals and *p* variables. When applying a dimension reduction to *q* components (q<p), the solution to the optimization problem defined in (1) is the largest eigenvalue λ of V, that is, Vw=λw. The eigenvalues of V are sorted in descending order as $\lambda_{1}>\ldots >\lambda_{q}$. The first principal component $w_{1}$ is the eigenvector of the unitary norm associated with the largest eigenvalue of V, that is, $\lambda_{1}$. Similarly, the second principal component $w_{2}$ is the eigenvector associated with $\lambda_{2}$, where $w_{1}$ and $w_{2}$ are orthogonal, that is, $w_{1}^{⊤}w_{2}=0$, and so on for the rest of the components.

In order to improve the interpretation, it is possible t o rotate the extracted components. Varimax is one of the most popular rotation methods and is applied to the component loadings (correlations between variables and factors); see details of the varimax method on p. 270 of [14]. The varimax orthogonal rotation tries to maximize the variance of the squared loadings in each factor, leading to large loadings for a few variables and the rest of variables with small loadings. Thus, it is possible to obtain a subset of variables that affect each component. Then, it is desirable that only one loading per observed variable is large in absolute value so that the variables are mainly related to exactly one component [9].

### 2.2. Disjoint Component Analysis

The DPCA is used as an alternative to classify variables and build clusters. The DPCA seeks to obtain disjoint principal components, that is, to say that the principal components should be highly correlated to some variables but not to others. In the PCA, the loading matrix B is orthogonal of p×q dimension. However, in the DPCA, in addition to it being an orthogonal matrix, $B=(b_{ij})$ must be a disjoint matrix, that is,

(i) For all *i*, there is only one *j*, such that $b_{ij}\neq 0$, with i∈{1,…,p} and j∈{1,…,q}.
(ii) For all *j*, there exists at least one *i*, such that $b_{ij}\neq 0$, with i∈{1,…,p} and j∈{1,…,q}.

The conditions (i) and (ii) above imply an increase of the constraints of the PCA optimization problem defined in (1). To obtain the disjoint components, the heuristic procedure presented in [15,16] can be used. Another procedure for computing disjoint components, based on particle swarm optimization, can be seen in [17], whereas an algorithm to compute disjoint components in three-way tables is presented in [18].

### 2.3. Functional Component Analysis

Unlike the PCA and DPCA, the FPCA considers data as functions, often over time [19,20,21]. The FPCA allows us to capture the variability of the phenomenon under investigation through its evolution over time. In the FPCA, each eigenvalue corresponds to an eigenfunction. The eigenfunctions describe the variability in the data set [22]. In the FPCA, the components are called harmonics.

In the PCA and DPCA, we have an n×p data matrix with *n* individuals and *p* original variables. Nevertheless, in the FPCA, we have *p* functions $x_{i}\left(t\right)$, for i=1,…,p, where the values of the data matrix $x_{it}$ are converted into functions $x_{i}\left(t\right)$, that is, the value of the variable *i* at time *t*, so one function is assigned to each original variable of the PCA [23].

In the multivariate context, a linear combination can be written as the dot product $w^{⊤}x$, where $w={\left(w_{1},\ldots ,w_{p}\right)}^{⊤}$ is a vector of weights and $x={\left(x_{1},\ldots ,x_{p}\right)}^{⊤}$ is a vector of variables. In the FPCA, linear combinations are represented as the dot product in a Hilbert space defined as

(2) $$L^{2}\left(T\right):\langle w,x\rangle =\int_{T}w(t)\;x\left(t\right)dt,$$

where *T* used in (2) is the time interval considered. In the FPCA, the sample covariance variance matrix is replaced by a bivariate function established by

(3) $$v\left(s,t\right)=\frac{1}{p}\sum_{i=1}^{p}x_{i}\left(s\right)x_{i}\left(t\right),$$

where the functions $x_{i}$ considered in (3) are centered.

Note that a functional principal component must satisfy the expression given by

(4) $$\int_{T}v(s,t)w\left(t\right)dt=\lambda w\left(t\right),$$

where λ is an eigenvalue and w is its corresponding eigenfunction. The equation given in (4) is the functional version of the matrix equation Vw=λw. There are several ways to solve (4); see, for example, [24,25]. Here, we follow the method outlined in [21]. Therefore, if the eigenvalues obtained by solving the equation given in (4) are sorted in descending order, as $\lambda_{1}>\ldots >\lambda_{q}$, the first functional principal component $w_{1}$ is the eigenfunction corresponding to $\lambda_{1}$. The second functional principal component $w_{2}$ is the eigenfunction associated with $\lambda_{2}$ of unitary norm and is orthogonal to $w_{1}$, and so on in such a way that the new functional principal component is orthogonal to the one calculated in (4), that is,

(5) $$\int_{T}w_{i}\left(t\right)\;w_{j}\left(t\right)dt=0,$$

for i<j. Thus, the score of the variable *i* in the component *k*, for k=1,…,q, is stated as

(6) $$f_{ik}=\int_{T}w_{k}\left(t\right)\;x_{i}\left(t\right)dt,$$

where the formulation defined in (6) represents the dot product between $w_{k}$ and $x_{i}$. Note that the expression given in (5) ensures that the functional components are orthogonal.

### 2.4. Algorithm and Computer Configurations

We have designed an algorithm that allows the multivariate methods presented in this work to sense changes in the data. This algorithm should be implemented in a software that, through the use of a sensor, would permit us to have an updated analysis. Algorithm 1 provides a summary of the methodology proposed in this study. The experimental analyses conducted here were carried out using a computer with the following characteristics: (i) OS: Windows 10 for 64 bits; (ii) RAM: 8 Gigabytes; and (iii) Processor: Intel Core i7-4510U 2–2.60 GHZ. The R software [26] was used for the PCA and FPCA, whereas the DPCA was implemented by the authors in C# based on the algorithm proposed in [15,16].
**Algorithm 1** Approach for sensing and updating the component analyses1:Update the local data warehouse with the data extracted from the website.2:Indicate the presence of new data in the local repository when the data warehouse, through a sensor (trigger), sends a signal to the software that performs the component analyses.3:Generate the score matrices and the corresponding loading matrices when the software receives the signal from the sensor and processes the DPCA through its C# implementation.4:Obtain the corresponding matrices to process the PCA and FPCA when the software communicates with R and sends the request to it.5:Produce the corresponding plots that serve as support for the component analyses when the software sends the request to R.6:Store the results of the component analyses in the local data warehouse by means of the software.7:End showing all the results of the updated component analyses in the graphical user interface using the software.

## 3. Results I

### 3.1. PCA and DPCA Results for the Number of COVID-19 Infected Cases

**Figure 1:**
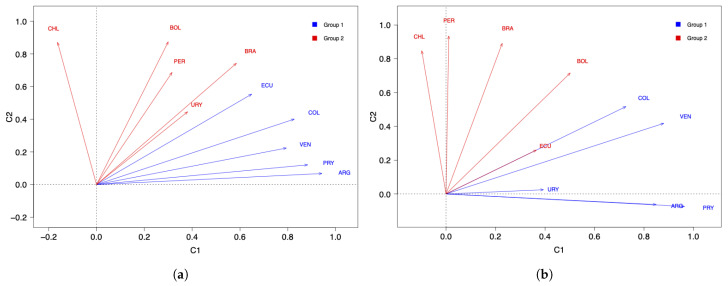
Space of countries for data of the number of COVID-19 (**a**) infected cases and (**b**) deaths.

A PCA was performed using the data in Table 1 with two components based on the correlation matrix. In this table, the columns show the countries and the rows show the number of COVID-19 infected cases (per million inhabitants) in each of the months from March 2020 to March 2021. In addition, a loading matrix was obtained but is not interpretable. Then, the varimax rotation was applied and a loading matrix, which is easier to interpret, was obtained; see Table 2. The percentage of variability explained by the model was 73.92%. Figure 1a shows the plot of the space of countries with the data of COVID-19 infected cases. From this figure, note that ARG, COL, PRY, and VEN are well represented by the first component, while BOL, BRA, CHI, PER, and URY are well represented by the second component. However, ECU has very similar loadings in both components.

To further improve the interpretation, a DPCA was carried out. Before performing the calculation of its components, as part of the preprocessing, a centering and scaling were applied to the matrix with the data of COVID-19 infected cases. Table 2 also reports the loading matrix with two disjoint components for the number of infected cases. The percentage of variability explained by the model was 69.27%. Of the variability, 4.65% was lost, but we gained in terms of interpretation as we can conclude that ECU is better represented in the first component. In summary, when using two components for the data on the number of COVID-19 infected cases, the South American countries are classified as: (Group 1) ARG, COL, PRY, VEN, and ECU; and (Group 2) BOL, BRA, CHI, PER, and URY.

Next, we performed a PCA with three components. A variability percentage of 87.07% was attained and once again the loading matrix obtained is not interpretable. Then, a varimax rotation was applied and no clearer interpretation was achieved, so we decided to use the DPCA again. When calculating three disjoint components, a variability percentage of 76.60% was obtained and 10.47% variability was lost, but an interpretable loading matrix was generated as observed in Table 3. In summary, when using three components for the data of the number of COVID-19 infected cases, the South American countries are classified as: (Group 1) ARG, COL, ECU, and VEN; (Group 2) BOL, BRA, CHI, and PER; and (Group 3) PRY and URY. Note that, when going from two to three components, one country came out of each of the two initial groups (PRY and URY, respectively) to form a third group.

### 3.2. PCA and DPCA Results for the Number of COVID-19 Deaths

Now, a similar analysis to that conducted in Section 3.1 was performed with the data in Table 4, where the columns show the countries and the rows show the number of COVID-19 deaths (per million inhabitants) in each of the months from March 2020 to March 2021.

Next, a PCA with two components was carried out using the correlation matrix. Then, a loading matrix was obtained but, once again, it is not interpretable. Thus, the varimax rotation was applied and a loading matrix that is easier to interpret was obtained in Table 5. The percentage of variability explained by the model was 69.75%. Figure 1b displays the plot of the space of countries with the data of deaths.

Notice that ARG, COL, PRY, URY, and VEN are represented by the first component, whereas BOL, BRA, CHI, and PER are represented by the second component. Similarly to the analysis of the COVID-19 infected cases, ECU has very similar loadings in both components. Disjoint components were calculated to improve the analysis. Again, centering and scaling were applied to the matrix with the data on deaths. Table 5 also presents the loading matrix with two disjoint components. The percentage of variability explained by the model was 64.75%, while 5% of variability was lost; however, we gain in terms of interpretation, concluding that ECU is better represented in the second component. In summary, when using two components for the data on the number of COVID-19 deaths, the countries are classified as: (Group 1) ARG, COL, PRY, URY, and VEN; and (Group 2) BOL, BRA, CHI, ECU, and PER.

A PCA with three components was also carried out and the model attained 85.28% of explained variability, but the loading matrix is not interpretable, so a varimax rotation was performed but the interpretation did not improve. When three disjoint components were computed, we obtained 74.78% of explained variability with 10.50% of variability being lost. Nevertheless, an interpretable loading matrix was achieved as can be seen in Table 6. In summary, when using three components for the data on the number of COVID-19 deaths, we have: (Group 1) ARG, COL, PRY, URY, and VEN; (Group 2) BRA, CHI, and PER; and (Group 3) BOL and ECU. Observe that, when going from two to three components, the first group keeps the same countries but the second group is subdivided into two others, where BOL and ECU come out of the second initial group to form a third group.

### 3.3. Analysis of the PCA and DPCA Results

**Figure 2:**
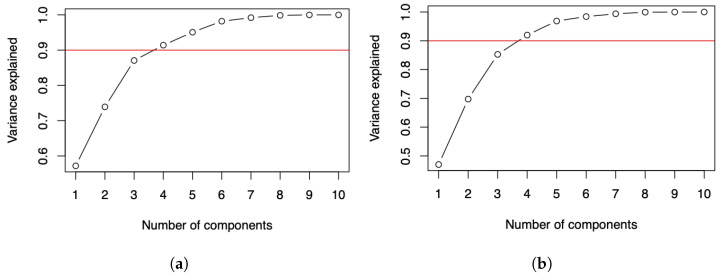
Cumulative variance plots of the number of COVID-19 (**a**) infected cases and (**b**) deaths.

For both data matrices reported in Table 1 and Table 4, the plots displayed in Figure 2a,b for the number of COVID-19 infected cases and deaths suggest two components if it is expected to explain about 70% of the variance, and three components if it is expected to explain about 90% of the variance. For more details about this criterion, see [27]. Some alternative criteria can be seen in [28,29].

**Figure 3:**
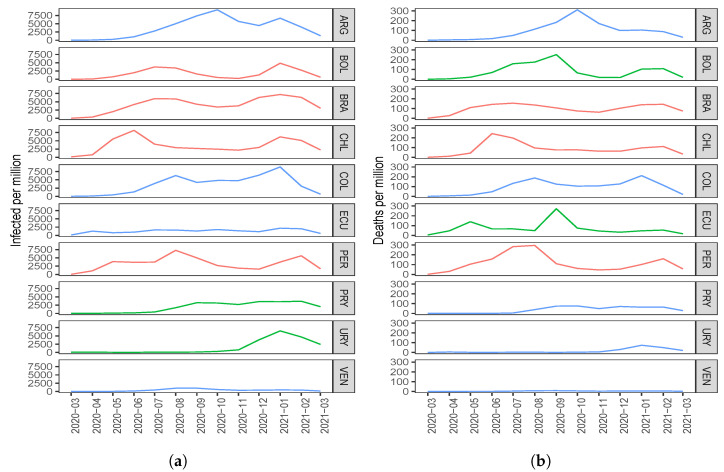
Plots of the number of COVID-19 (**a**) infected cases and (**b**) deaths for the indicated country and month.

Regarding the number of COVID-19 infected cases with two components, the countries with the highest score in the first component (C1) are ARG, PRY, and COL. The countries with the highest scores in the second component (C2) are BOL, CHI, and BRA; see Figure 1a. When comparing this clustering with Figure 3a, interpreting the components and considering the countries with the highest scores, note that the component C1 groups countries that took time to reach the first peak of the pandemic, while the C2 component groups countries that reached both peaks of the pandemic at similar times.

About the number of COVID-19 deaths with two components, the countries with the highest scores in C1 are PRY, ARG, and VEN. The countries with the highest scores in C2 are PER, BRA, and CHI; see Figure 1b. When comparing this clustering with Figure 3b, interpreting the components and considering the countries with the highest scores, observe that C1 groups countries that had a small number of deaths after the December 2020 holidays, while C2 groups countries that had an increase in the number of deaths after these holidays.

The previous sections showed the results of the classification of countries for the number of COVID-19 infected cases and deaths with two and three components. Despite the fact that two components are suggested in both situations, we decided to also perform the calculations with three components in order to include in this work an analysis with a higher percentage of variability (using a third principal component).

In relation to the number of COVID-19 infected cases, adding a third component, we gain 13.15% and 7.33% in explained variability with standard and disjoint components, respectively. Regarding the number of deaths, when going from two to three components, we gain 15.53% and 10.03% in variability with standard and disjoint components, respectively. In general, when using three principal components, there is an explained variability close to 90% for both the number of infected and the number of deaths due to COVID-19. Figure 3a,b represent Table 1 and Table 4 graphically, respectively. Countries colored in blue, red, and green are grouped by components C1, C2 and C3, respectively; see Table 3 and Table 6. When comparing Figure 3a,b with the results obtained using two components, it is hard to detect similarities and differences in the ten South American countries. However, when using three components, we consider that a better classification is obtained.

Regarding the number of COVID-19 infected cases with three components, in C1 we have ARG, COL, VEN, and ECU; in C2, we have BOL, BRA, PER, and CHI; whereas in C3, we have PRY and URY. By observing Figure 3a, we can interpret that C1 groups those countries that have had similar behavior since December 2020. Note that ARG has a peak and a fall similar to that which happens with COL. In addition, ECU and VEN have similar behavior to each other, with small values in the number of infected. We can interpret component C2 as being those countries that had similar behavior in both peaks; even those times at which the peaks occurred, the time that elapsed between them was also similar. In the case of component C3, it groups those countries that had few infected cases at the beginning of the pandemic and that reached a peak after the December 2020 holidays. These similarities between PRY and URY are detected by component C3, but with only two components this would not have been detected.

About the number of COVID-19 deaths with three components, we have PRY, VEN, COL, ARG, and URY in C1; BRA, PER, and CHI in C2; and in C3, BOL and ECU. From Figure 3b, we can note that component C1 groups the countries with the smallest number of deaths, which are PRY, VEN, and URY. In this first component, we also identify ARG and COL with a smaller number of deaths at the beginning and at the end of the study period. We can interpret component C2 as those countries that had similar behavior in both peaks, the first between June 2020 and July 2020, and the second peak that was reached after the December 2020 holidays. Furthermore, observe that C3 groups those countries that reached their first peak in September 2020 and had an increase in the number of deaths after the holidays. The similarities between BOL and ECU are detected when C3 is considered, but not with C2.

Note that, when using the PCA and DPCA, both for the number of COVID-19 infected cases and deaths, the components are based on the number of peaks and the time in which these peaks are reached. In addition, observe that the beginning, duration, and end of the waves are considered in the groupings.

As mentioned, the PCA enables us to classify the countries analyzed in this study, with the components being latent countries. The DPCA was performed to contrast what was obtained with the PCA obtaining results consistent. The grouping of the countries, according to the number of COVID-19 infected cases and deaths (in both cases per million inhabitants), was carried out to comparatively determine similarities and differences among the countries during the time considered in our investigation (13 months).

### 3.4. k-Means Cluster Analysis for Countries

**Figure 4:**
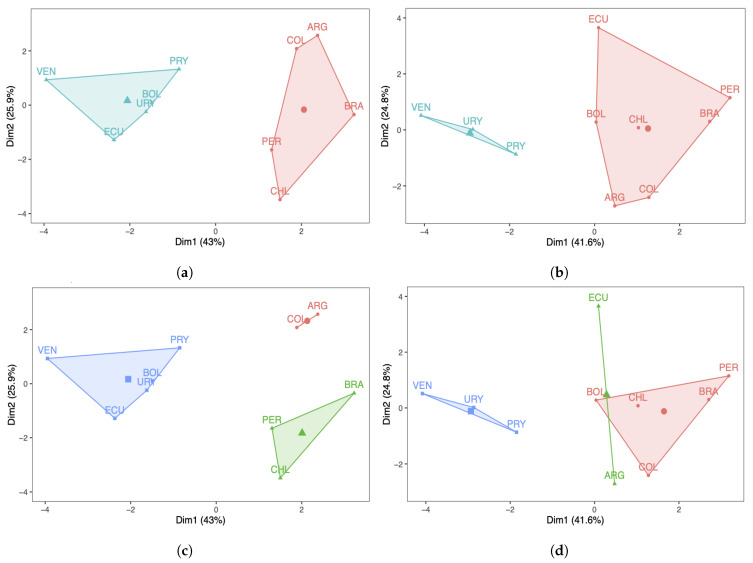
Cluster plots with (**a**,**b**) k=2 and (**c**,**d**) 3 of the number of COVID-19 (**a**,**c**) infected cases and (**b**,**d**) deaths.

Next, a k-means analysis was carried out for the South American countries under study. Figure 4a,b show the results with two clusters, whereas Figure 4c,d display the results with three clusters. Regarding the number of COVID-19 infected cases with two clusters, note that in Figure 4a the countries are grouped in a different way when compared with the PCA/DPCA grouping using two components. From Figure 4b, we can conclude that the same occurs with respect to the number of deaths, that is, the results of the clustering with k-means differ from the clustering obtained with the PCA/DPCA. When comparing the three clusters of the k-means analysis with the three components of the PCA/DPCA, for the number of COVID-19 infected cases and their deaths, the results obtained are more similar than when comparing the two clusters of k-means and the two components of the PCA/DPCA. However, there are also differences. In this work, we consider that the results obtained with the PCA are more reliable and better reflect reality than the k-means analysis for the following reasons:

(i) The PCA reduces the dimensionality of the data, grouping the ten original countries into two or three latent countries. These latent countries (components) are linear combinations of the original countries. The PCA allows us to interpret the components obtained considering the original variables (countries) with the highest score in each of the components.

(ii) In the k-means analysis, we must indicate, from the beginning, the number of groups to be constructed, minimizing the squared distance of the variables (countries) from the centroid. Furthermore, k-means is an association method, which forms groups through their similarity. However, the PCA works with the full data set, maximizing the explained variance to obtain the components, one by one, trapping as much information as possible that it finds in the data.

## 4. Results II

### 4.1. FPCA Results for the Number of COVID-19 Infected Cases

**Figure 5:**
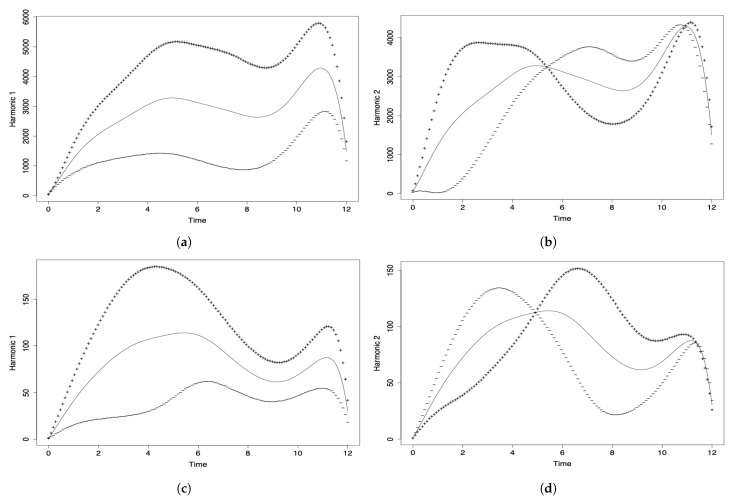
Harmonic components (**a**,**c**) 1 and (**b,d**) 2 of the FPCA for data on the number of COVID-19 (**a**,**b**) infected cases, and (**c**,**d**) deaths.

Different to the PCA and DPCA, the FPCA allows us to capture the variability of the phenomenon under investigation through its evolution over time. In the FPCA, we associate a proper or harmonic function with each eigenvalue, which describes the main modes of variation existing in the data set. In Figure 5a,c, month 0 corresponds to March 2020, month 1 to April 2020, month 2 to May 2020, and so on until month 12, which corresponds to March 2021.

In Figure 5a, the first component (solid line) reflects the general evolution of the number of COVID-19 infected cases reported per month throughout the pandemic in all the countries considered. The greatest influence can be seen from months 5 to 11 (August 2020 to February 2021). The decline at the end of the curve is due to the fact that the data for March 2021 are only considered until the middle of the month. The second principal functional component (solid line) is shown in Figure 5b and it agrees with the variation detected in the first principal component. This second component only contributes 21.3% of the explained variability and is not as important as the first one.

**Figure 6:**
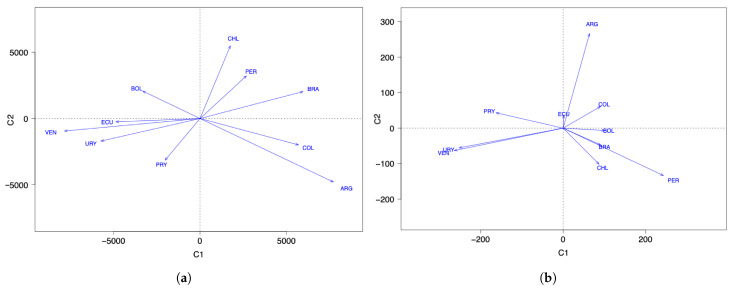
FPCA space of countries for data on the number of COVID-19 (**a**) infected cases and (**b**) deaths.

Table 7 and Figure 6a show the behavior of the countries over the course of the pandemic. Note that ECU, URY, and VEN have similar behavior, which is in contrast to BRA. In addition, the behavior of ARG and CHI differs from the rest. However, ARG and BOL demonstrate the opposite behavior from each other in the evolution of the number of new COVID-19 cases reported. We must consider that the number of new cases depends on the number of PCR tests applied, which might explain why some countries have a low rate of new cases. The position to the left of the origin in Figure 6a can be due to two situations, either: (i) the health policies are managed correctly; or (ii) insufficient detection PCR tests are carried out. A country in the first situation is URY, which is an example of correct management at the beginning of the pandemic that then ceased to be so.

### 4.2. FPCA Results for the Number of COVID-19 Deaths

Figure 5c shows the first functional component (solid line), reflecting the temporal evolution of the number of deaths per month and per million inhabitants globally (all countries considered simultaneously). The hightest variability is due to the contribution of the months of June to September 2020. Then, we see a decrease and a rise that can be attributed to the holiday season. As in the previous analysis of the number of COVID-19 infected cases, the decline at the end of the curve is due to the fact that the data for March 2021 are only considered until the middle of the month. The behavior of the second principal functional component (solid line) is similar to that of the first component (see Figure 5b), not providing more information on the temporal evolution of the event. Its percentage of variability explained is much less than for the first principal component.

Table 8 reports the loadings for the first two components and Figure 6b shows the relative positions of the countries considered with respect to the number of COVID-19 deaths per month and per million inhabitants throughout the pandemic. Considering the projections on the first main axis, we see that the countries with the smallest number of deaths (per month and per million) are PRY, URY, and VEN, which are the least affected countries with respect to the number of deaths, but ECU is the average. Forming a group, we have BOL, BRA, CHI, and COL, which means that their behavior regarding the number of deaths is similar, with PER being in the position furthest to the right of the axis, indicating that it is the country hardest hit (in percentage terms) by the pandemic. Note that the officially reported number of deaths due to COVID-19 may differ significantly from the true number of deaths, as has been observed in several countries in the region.

### 4.3. k-Means Cluster Analysis for Time

To complement the component analysis conducted, we also carried out a k-means analysis for time with three clusters. First, we show the results regarding the number of COVID-19 infected cases. Table 9 reports the scores of the countries in each of the clusters and Table 10 shows the membership of each studied month to each cluster.

Table 9 and Table 10 allow us to conclude that, from March to June 2020 (also March 2021), CHI is the country with the most infections, followed by PER and BRA. In addition, from July to November 2020, ARG is the country with most infections, followed by COL, BRA, and PER. From December 2020 to February 2021, BRA is the country with the most infections, followed by COL, ARG, and URY.

The k-means analysis is consistent with the results of the FPCA, since Cluster 1 corresponds to the months in which the two functional components show a growth in the number of COVID-19 infected cases. Cluster 2 is associated with the months in which the functional components show a decrease. Cluster 3 is related to the months in which there is a more pronounced growth than in Cluster 1 (as a consequence of the end of the year holidays).

Now we show the results regarding the number of COVID-19 deaths. Table 11 reports the scores of the countries in each of the clusters, whereas Table 12 presents the membership of each studied month to its cluster. Table 11 and Table 12 allow us to conclude that, from March to May 2020 (also March 2021), and in November and December 2020, BRA is the country that presented most deaths, followed by ARG, PER, ECU, and COL. From June to August 2020, and in January and February 2021, PER had the largest number of deaths, followed by CHI, BRA, COL, and BOL. In September and October 2020, ARG presented more deaths, followed by ECU, BOL, and COL.

The three clusters detected by the k-means method correspond to the months in which the functional components show growth in the number of deaths, a more attenuated growth, and a decrease. Thus, Cluster 2 corresponds to the months in which the main functional components show a marked growth in the number of deaths. Cluster 3 is related to the months in which the functional components indicate a less accelerated growth in the number of deaths compared to Cluster 2. Note that Cluster 1 is associated with the months in which there is a decrease in the number of deaths due to COVID-19.

## 5. Conclusions

The behavior of the number of infected cases with COVID-19, and the number of deaths due to this disease, can vary in the countries for different reasons: (i) political and economic decisions; (ii) health infrastructure; (iii) the discipline of the people; (iv) the environment; and (v) the spread rates of the different strains of the virus, among other factors.

Principal component analyses based on recent and modern methods were carried out to study the relationship among the aforementioned variables in ten South American countries. The countries considered were: Argentina, Bolivia, Brazil, Chile, Colombia, Ecuador, Peru, Paraguay, Uruguay, and Venezuela. A k-means analysis was conducted to compare our principal component analyses, which resulted in general consistent.

By using a principal component analysis with varimax rotation and a disjoint component analysis, we report the following results: with two components, and considering the number of COVID-19 infected cases, there are two groups of countries, with Argentina, Colombia, Ecuador, and Venezuela in one group, while Bolivia, Brazil, Chile, Peru, and Uruguay are in another group. When increasing the number of components to three, Paraguay and Uruguay moved away from the other countries and formed a third group.

When considering the number of COVID-19 deaths and two components, we established two groups formed by Argentina, Colombia, Paraguay, Uruguay, and Venezuela in one group, while Bolivia, Brazil, Chile, Ecuador, and Peru were in another group. However, with three components, Bolivia and Ecuador moved away from the other countries and formed a third group.

In the case of using functional components, we conclude that both components showed a general evolution of the number of COVID-19 infected cases reported per month throughout the pandemic, in all the countries considered. The largest values were detected in the months of July, August and September 2020 on the one hand, and in the months of January and February 2021 on the other hand. Regarding the relative positions of the countries, Ecuador, Uruguay, and Venezuela had very similar behavior, and Brazil, Chile, and Peru also behaved similarly to each other. Another group was made up of Argentina and Colombia, while Bolivia and Paraguay were far from the rest of the countries and between them.

Considering the number of deaths using functional components, there are peaks in the months of September 2020 on the one hand, and January and February 2021 on the other hand, which is similar to what happened with the number of COVID-19 infected cases. The countries with the smallest number of deaths were Paraguay, Uruguay, and Venezuela, whereas a second group was formed by Bolivia, Brazil, and Chile, with their behavior regarding the number of deaths being similar and greater than the average. In the case of Peru, this country was the most affected by the pandemic, while Ecuador was within the average; Argentina was a little more affected than Ecuador, but less than Chile. It should be borne in mind that the officially reported number of deaths due to COVID-19 may differ significantly from the number of true deaths, as has been observed in several South American countries.

Data related to COVID-19 can change over time. Therefore, the conclusions to be obtained from them, after statistical analyses, are sensitive to these changes. As mentioned, the data were obtained from the repository for the 2019 Novel Coronavirus Visual Dashboard operated by the Johns Hopkins University Center for Systems Science and Engineering (JHU CSSE) [30], which keeps the data updated, particularly the number of COVID-19 infected cases and the number of deaths due to this disease. These data were also used in [31]. Note that the number of confirmed cases is less than the number of true cases because not all people are tested with PCR. This means that the number of confirmed cases depends on how many PCR tests a country applies.

When looking at Figure 3, we can see that some countries, such as Bolivia, Brazil, Chile, and Peru, reacted late in controlling the pandemic because they were the countries to reach a first peak early with respect to the number of COVID-19 infected cases and deaths. Figure 3 also shows that countries such as Uruguay, Paraguay, and Venezuela had better control of the pandemic since the peaks of infected cases and deaths due to COVID-19 were reached some time after the aforementioned countries.

Despite the fact that some countries already knew that they had COVID-19 infected cases in their territory since the beginning of February 2020, it was in mid-March 2020 when the governments made the first decisions to control the spread of the virus. With full knowledge of what was happening in Asia and Europe, some South American countries minimized the impact that COVID-19 would have on the economy [2,3] and on people’s health. Other countries were a little more cautious and took strong measures from mid-March 2020, such as closing borders and confining citizens. However, it must also be said that, regardless of the policies taken by the governments [4], the climate, the limitations of the health systems (infrastructure, personnel), the behavior of citizens, among other factors, also affected the variables of this study. By classifying countries into groups, we can comparatively study the damage caused by the COVID-19 pandemic, analyzing peaks and waves.

We consider that a better interpretation and classification of the countries is obtained using three principal components, leading to the following conclusions:

(i) For the number of COVID-19 infected cases, the components C1, C2, and C3 can be interpreted as: C1 grouped those countries that had similar behavior since December 2020; C2 grouped those countries with similar behavior throughout the entire period of analysis; and C3 grouped those countries that had few infected cases at the beginning of the pandemic and reached a peak after the December 2020 holidays.

(ii) For the number of COVID-19 deaths, the components C1, C2, and C3 can be interpreted as: C1 grouped those countries with the smallest number of deaths, at the beginning and at the end of the study period; C2 grouped those countries with similar behavior in both peaks; and C3 grouped the countries that reached their first peak in September 2020 and reached a second peak after the December 2020 holidays.

For further research, this study can be replicated for other regions of the planet such as the USA, Europe, and Asia. A study can also be conducted to implement alternative criteria for selecting the correct number of components [28,29].

## Figures and Tables

**Table 1:** Data matrix of the number of COVID-19 infected cases per million inhabitants for the indicated month and country.

Month	ARG	BOL	BRA	CHI	COL	ECU	PER	PRY	URY	VEN
2020-03	23.320	9.168	26.887	148.671	17.808	126.961	32.300	9.112	97.303	4.748
2020-04	74.653	90.807	383.281	777.248	110.077	1286.283	1089.142	28.181	87.802	6.963
2020-05	274.872	755.160	2011.963	5537.081	449.583	802.809	3866.934	100.945	51.820	41.393
2020-06	1054.943	1990.654	4173.853	8152.400	1345.499	982.482	3661.822	173.150	32.533	151.992
2020-07	2804.951	3732.537	5929.842	3990.017	3884.646	1639.341	3708.588	437.013	94.426	448.096
2020-08	5010.048	3410.341	5860.889	2932.538	6280.816	1610.378	7269.051	1727.860	95.286	990.088
2020-09	7373.831	1603.095	4246.639	2681.757	4217.234	1319.497	4992.196	3238.125	129.832	998.524
2020-10	9202.697	552.215	3409.259	2472.506	4805.250	1765.282	2681.440	3144.328	310.331	594.001
2020-11	5699.844	252.801	3764.939	2170.254	4768.245	1388.254	1891.010	2697.360	786.763	365.067
2020-12	4446.897	1320.651	6304.567	2993.796	6406.262	1123.783	1595.511	3576.292	3817.801	392.603
2021-01	6675.956	4858.291	7192.142	6179.886	8885.288	2171.731	3733.550	3546.429	6511.452	470.146
2021-02	3985.461	2756.354	6334.831	5101.220	3081.706	2002.317	5629.776	3679.898	4679.701	428.651
2021-03	1379.088	638.395	3062.888	2266.347	673.508	605.678	1703.270	2038.407	2444.060	147.877

**Table 2:** Loading matrices for data of the number of COVID infected cases with two components (C1 and C2) for the indicated country and method.

	Varimax		DPCA	
Country	C1	C2	C1	C2
ARG	0.943	0.068	0.484	0.000
BOL	0.300	0.874	0.000	0.519
BRA	0.585	0.743	0.000	0.519
CHI	−0.163	0.871	0.000	0.415
COL	0.827	0.401	0.466	0.000
ECU	0.649	0.554	0.417	0.000
PER	0.316	0.687	0.000	0.416
PRY	0.883	0.121	0.438	0.000
URY	0.381	0.446	0.000	0.340
VEN	0.794	0.224	0.428	0.000

**Table 3:** Loading matrix for data on the number of COVID infected cases with three components (C1, C2, and C3) for the indicated country using the DPCA.

	DPCA		
Country	C1	C2	C3
ARG	0.524	0.000	0.000
BOL	0.000	0.541	0.000
BRA	0.000	0.525	0.000
CHI	0.000	0.446	0.000
COL	0.515	0.000	0.000
ECU	0.472	0.000	0.000
PER	0.000	0.483	0.000
PRY	0.000	0.000	0.707
URY	0.000	0.000	0.707
VEN	0.487	0.000	0.000

**Table 4:** Data matrix of the number of COVID-19 deaths per million inhabitants for the indicated month and country.

Month	ARG	BOL	BRA	CHI	COL	ECU	PER	PRY	URY	VEN
2020-03	0.598	0.514	0.946	0.625	0.315	4.25	0.912	0.42	0.288	0.105
2020-04	4.228	4.802	27.309	11.245	5.446	46.759	30.97	0.98	4.608	0.456
2020-05	7.103	21.505	109.652	43.261	12.695	139.319	104.785	0.14	1.44	1.46
2020-06	16.992	69.389	142.453	242.41	47.07	66.258	156.833	0.84	1.44	1.3
2020-07	49.472	158.827	154.691	197.164	133.072	66.599	283.394	4.481	2.304	3.982
2020-08	113.22	175.617	135.991	95.837	187.824	48.405	296.222	38.837	2.592	7.807
2020-09	183.136	251.691	106.187	75.957	124.52	272.006	109.426	74.447	1.152	8.511
2020-10	311.202	65.108	74.954	76.687	104.477	74.534	61.111	76.691	2.88	5.981
2020-11	170.991	19.877	62.272	62.93	107.144	44.833	45.854	49.35	5.472	3.489
2020-12	99.897	17.817	102.695	62.666	126.704	32.477	53.289	70.943	29.943	4.615
2021-01	104.633	103.999	139.043	96.464	211.664	46.757	101.482	63.931	73.409	5.663
2021-02	88.304	108.795	143.198	110.903	113.657	53.959	159.924	64.912	49.515	5.453
2021-03	30.843	20.133	73.927	33.165	19.789	16.664	56.533	28.879	20.151	1.934

**Table 5:** Loading matrices for data on the number of COVID deaths with two components for the indicated country and method.

Country	Varimax		DPCA	
ARG	0.849	−0.063	0.438	0.000
BOL	0.502	0.716	0.000	0.473
BRA	0.227	0.891	0.000	0.512
CHI	−0.098	0.847	0.000	0.452
COL	0.727	0.517	0.476	0.000
ECU	0.365	0.26	0.000	0.247
PER	0.011	0.935	0.000	0.499
PRY	0.964	−0.074	0.509	0.000
URY	0.394	0.025	0.259	0.000
VEN	0.880	0.418	0.505	0.000

**Table 6:** Loading matrix for data on the number of COVID deaths with three components (C1, C2, and C3) for the indicated country using the DPCA.

	DPCA		
Country	C1	C2	C3
ARG	0.438	0.000	0.000
BOL	0.000	0.000	0.707
BRA	0.000	0.594	0.000
CHI	0.000	0.566	0.000
COL	0.476	0.000	0.000
ECU	0.000	0.000	0.707
PER	0.000	0.571	0.000
PRY	0.509	0.000	0.000
URY	0.259	0.000	0.000
VEN	0.505	0.000	0.000

**Table 7:** Loading matrix for data on the number of COVID infected cases with two components (C1 and C2) for the indicated country using the FPCA.

	FPCA	
Country	C1	C2
ARG	7707.767	−4791.927
BOL	−3319.535	2075.921
BRA	5959.808	2033.667
CHI	1755.468	5492.041
COL	5692.081	−1991.679
ECU	−4878.641	−249.683
PER	2686.448	3222.517
PRY	−2023.569	−3145.071
URY	−5732.866	−1702.523
VEN	−7846.961	−943.263

**Table 8:** Loading matrix for data on the number of COVID-19 deaths with two components (C1 and C2) for the indicated country using the FPCA.

	FPCA	
Country	C1	C2
ARG	64.104	266.481
BOL	100.787	−6.533
BRA	91.109	−47.293
CHI	86.967	−101.372
COL	90.490	61.045
ECU	1.621	36.494
PER	243.162	−133.709
PRY	−162.396	43.453
URY	−252.150	−55.442
VEN	−263.694	−63.125

**Table 9:** Center of the listed cluster for the number of COVID-19 infected cases in the indicated country using the k-means method.

Cluster	ARG	BOL	BRA	CHI	COL	ECU	PER	PRY	URY	VEN
1	561.375	696.837	1931.774	3376.349	519.295	760.843	2070.694	469.959	542.704	70.595
2	6018.274	1910.198	4642.314	2849.414	4791.238	1544.550	4108.457	2248.937	283.328	679.155
3	5036.105	2978.432	6610.513	4758.301	6124.419	1765.944	3652.946	3600.873	5002.985	430.467

**Table 10:** Membership of the listed cluster for the number of COVID-19 infected cases in the indicated month using the k-means method.

Month	20-03	20-04	20-05	20-06	20-07	20-08	20-09	20-10	20-11	20-12	21-01	21-02	21-03
Cluster	1	1	1	1	2	2	2	2	2	3	3	3	1

**Table 11:** Center of the listed cluster for the number of COVID-19 deaths in the indicated country using the k-means method.

Cluster	ARG	BOL	BRA	CHI	COL	ECU	PER	PRY	URY	VEN
1	247.169	158.400	90.571	76.322	114.499	173.270	85.269	75.569	2.016	7.246
2	52.277	14.108	62.800	35.649	45.349	47.384	48.724	25.119	10.317	2.010
3	74.524	123.325	143.075	148.556	138.657	56.396	199.571	34.600	25.852	4.841

**Table 12:** Membership of the listed cluster for the number of COVID-19 deaths in the indicated month using the k-means method.

Month	20-03	20-04	20-05	20-06	20-07	20-08	20-09	20-10	20-11	20-12	21-01	21-02	21-03
Cluster	2	2	2	3	3	3	1	1	2	2	3	3	2

## Data Availability

The full data set used in this investigation can be downloaded from https://ourworldindata.org/coronavirus (accessed on 12 June 2021).
